# Positional nystagmus is observed in the vast majority of healthy individuals

**DOI:** 10.1007/s00405-024-08453-y

**Published:** 2024-02-01

**Authors:** Mads Bolding Rasmussen, Rasmus Sørensen, Dan Dupont Hougaard

**Affiliations:** 1 Department of Clinical Medicine, Aalborg University, Aalborg, Denmark; 2 Balance and Dizziness Centre, Department of Otorhinolaryngology, Head and Neck Surgery and Audiology, Aalborg University Hospital, Havrevangen 1, Aalborg, Denmark

**Keywords:** Benign paroxysmal positional vertigo, Positional nystagmus, Healthy population, Mechanical rotational chair

## Abstract

**Introduction:**

Benign paroxysmal positional vertigo (BPPV) is a vestibular disease characterized by brief  positional vertigo. When examined, characteristic patterns of positional nystagmus (PN) are found with specific head position changes. Previous studies have shown a high prevalence of PN among vestibular healthy subjects. Considering the current diagnostic criteria of BPPV and the potentially high prevalence of PN in healthy individuals, this raises the question of potential over diagnosing BPPV, if diagnostics are based exclusively upon objective findings. This study aims to determine the prevalence of PN within a healthy, adult population and furthermore include a characterization of the PN observed.

**Methods:**

This is a prospective cross-sectional study. 78 subjects were included. The subjects underwent four standardized positional tests for BPPV in a mechanical rotational chair while using a VNG-goggle to monitor and record eye movements.

**Results:**

Positional nystagmus was recorded in 70.5% (55/78) of the subjects. Of the 55 subjects, who presented with PN, 81.8% (45/55) had upbeating PN. The 95th percentile of the maximum a-SPV was found to be 10.4 degrees per second, with a median of 4. Five subjects (6.4%) in total presented with PN mimicking BPPV.

**Conclusion:**

This study found PN to be a common finding within a healthy, adult population based on the high prevalence of PN in the study population. Upbeating PN mimicking posterior canalolithiasis was found in numerous subjects. The authors recommend a cautious approach when diagnosing BPPV, especially in cases of purely vertical PN (without a torsional component) and if no vertiginous symptoms are present during Dix-Hallpike and Supine Roll Test examinations.

## Introduction

### Benign paroxysmal positional vertigo

Benign paroxysmal positional vertigo (BPPV) is a vestibular disease consisting of brief attacks of rotational vertigo. When examined, characteristic patterns of positional nystagmus (PN) are found with specific head position changes. It is the most common vestibular disease accounting for 17 to 42% of patients with vertiginous symptoms [1, 2]. BPPV predominantly affects women and studies have shown an estimated lifetime prevalence of 2.4% with the highest incidence occurring through the ages of 50 to 70 years and a clear tendency of an increasing prevalence with increasing age [3]. Although BPPV is a benign disease, it may have severe consequences in terms of quality of life (QoL) [4].

BPPV originates from one or both vestibular organ(s) which consist(s) of three semicircular canals (SCCs) as well as two otolithic organs, the saccule and the utricle bilaterally [1].

The most accepted hypothesis regarding the etiology of BPPV is that otoconial debris dislodge from the utricle into the endolymph of one or several of the SCCs. If otoconia enter the SCCs, these structures become sensitive to gravity. Head movements in the plane(s) of the involved SCC(s) make the otoconia move, bends the cilia of the cupula within the affected SCC(s), and increase the neural activity. The vestibular ocular reflex (VOR) is activated with compensatory eye movements (nystagmus) resulting in a false sensation of movement perceived as vertigo [2, 5]. When otolithic debris is moving freely within the SCC, the condition is termed canalolithiasis (CAN). If instead the debris is attached to the cupula, the condition is termed cupulolithiasis (CUP) [1].

### Diagnostic criteria

Prevailing diagnostic criteria of BPPV are not exclusive and are therefore also not completely unanimous. The Bárány Society and the American Academy of Otolaryngology, Head and Neck Surgery (AAO-HNS) have set up a series of precise diagnostic criteria that must be fulfilled to diagnose BPPV. These two sets of diagnostic criteria are not completely identical. Even though both sets of criteria require the presence of PN during specific head position changes, only the AAO-HNS criteria require the patient to experience positional vertigo during the diagnostic examinations. The Bárány Society instead include a history of recurrent attacks of vertigo or dizziness when changing head positions (classical BPPV history) [2, 6]. Additionally, a recent study has found a relatively high prevalence of PN in healthy patients without any classical BPPV history [7].

### Mechanical rotational chairs

Studies have shown that usage of mechanical rotational chairs (MRCs) allows more precise diagnostics and more effective treatments of patients with refractory BPPV i.e., lateral, anterior, multi-canal, refractory posterior BPPV as well as patients who cannot cooperate to traditional diagnostics and treatments on an examination bed [8].

The Thomas Richard-Vitton (TRV)-chair is a bi-axial (allows 360-degree rotations in the yaw- and pitch axes) non-motorized MRC that is operated by hand. The design of this MRC ensures that the examiner can position and move the patient in fixed planes aligned with the anatomical positions of the paired SCCs [9].

### Prevalence of positional nystagmus in a healthy population

Previous studies have shown a high prevalence of PN (between 48 to 100%) amongst healthy individuals i.e., a population of individuals with neither vertiginous symptoms nor previous or current disease(s) and/or condition(s) that may cause PN [7, 10]. Although some of these previous findings are similar to the PN associated with BPPV, PN characteristics in a healthy population differ on several parameters: 1) the a-SPV tends to be lower (indicating less severe nystagmus), 2) the PN tends to be persistent throughout the examination, 3) torsional nystagmus seems to be less common [7]. Considering the current diagnostic criteria of BPPV and the potentially high prevalence of PN in healthy individuals, this raises the question of whether there might be a tendency of over diagnosing BPPV, if diagnostics are based exclusively on objective findings, in particular by a novice examiner.

### Aim of study

Primary objective was to determine the prevalence of PN within a cohort of healthy Danish adults. Secondary objectives included: 1) characterization of the observed PN, and 2) identification of additional nystagmus parameters that might aid and refine the current diagnostic criteria of BPPV.

## Methods

This study was done as a prospective cross-sectional study.

### Population and screening

A total of 84 healthy adults were recruited through social media groups of Aalborg University in Denmark and word of mouth. 18 years of age or older was the sole inclusion criteria.

Exclusion criteria included previous or current diagnoses of BPPV, other inner ear disease(s), neurological disease(s), impaired eye muscle motility, bodyweight above 150 kg or inability to participate in the examinations carried out with the TRV-chair (T-MRC) (Interacoustics®, Middelfart, Denmark). Additional exclusion criteria included diagnoses of cerebral aneurism as well as cerebral hemorrhage one month prior to the examination.

Subjects underwent a series of clinical examinations to rule out any conditions potentially influencing the test results prior to examinations in the T-MRC. Pre-examination screening included a test of eye muscle motility, a Vestibular Romberg’s Test (with subjects standing on a foam mattres), a skew deviation test, and a complete Video Head Impulse Test including all six SCCs (vHIT, Otometrics®, Taastrup, Denmark). A pathological Romberg’s test, a pathological skew deviation test, a pathological vHIT test or clinical findings of reduced eye-motility led to exclusion. Of 84 recruited subjects, two did not show up for examinations, two had a pathological Romberg’s test, and one a pathological vHIT. Furthermore, one subject presented with spontaneous nystagmus in the T-MRC. This led to exclusion of this subject. Therefore, following screening, a total of 78 subjects were eligible for study inclusion.

### Video head impulse testing

With vHIT, which examines the function of VOR of all individual SCCs, the test conditions were standardized in the following way: (1) subjects were placed on the same non-revolving chair one meter away from a fixation dot on a wall, (2) markings on the floor indicated the exact position for the chair being used for both horizontal and vertical SCC-testing, (3) the same lighting was used during every test, (4) both examiners were given thorough instructions on how to perform the test adequately and how to minimize the amount of artefacts and noise added during the actual testing, and (5) both examiners performed pre-trial testing until an experienced examiner approved their test skills.

Prior to each examination, calibration was performed according to the manufacturer’s recommendations. Initially, lateral SCC-testing was performed by application of head impulses in the horizontal plane with the subject’s head bend approximately 30 degrees forward. Subsequently, the left anterior and right posterior (LARP) SCCs and the right anterior and left posterior (RALP) SCCs were examined by using the 2D vHIT method [11]. While doing the actual vHIT testing, both examiners ensured that head impulses were fast, abrupt, unpredictable, with high acceleration, and with low amplitude. During testing, a minimum of 15 head impulses (approved by the accompanying software) were applied to each and all six SCCs. Post-test interpretation of vHIT results was done according to the recommendations by Hougaard et al. by both examiners [11]. A pathological vHIT examination of minimum one SCCs excluded subjects from further examinations, as this was considered a sign of peripheral disease involving either the vestibular nerve(s) and/or structures within the inner ear.

The questionnaire termed the “Dizziness Handicap Inventory”, was completed by all subjects to determine if subjects experienced either no dizziness or a mild, moderate or even severe dizziness handicap. A total DHI-score of 14 or above excluded subjects from the study, as this score would indicate vestibular or central pathology.

All subjects received written and oral information about the research project. Written consent was obtained from all subjects prior to enrolment in the study.

### Setting and examinations

All examinations took place at the Balance & Dizziness Centre at Aalborg University Hospital in Denmark. The same two examiners did all pre-inclusion testing as well as all examinations related to the study. Subjects were randomized according to examiner and order of positional tests being performed. Before any positional testing was performed, the two examiners made sure that neither spontaneous nystagmus (with and without fixation) nor gaze induced nystagmus was present. The four traditional positional examinations included in the diagnostics of BPPV as well as a Supine Position test were carried out in random order. These included a deep left and right Dix Hallpike-test (DH-test), a Supine Position test as well as a left and right Supine Roll Test (SRT). All five positions were retained for 60 seconds.

**Fig. 1:**
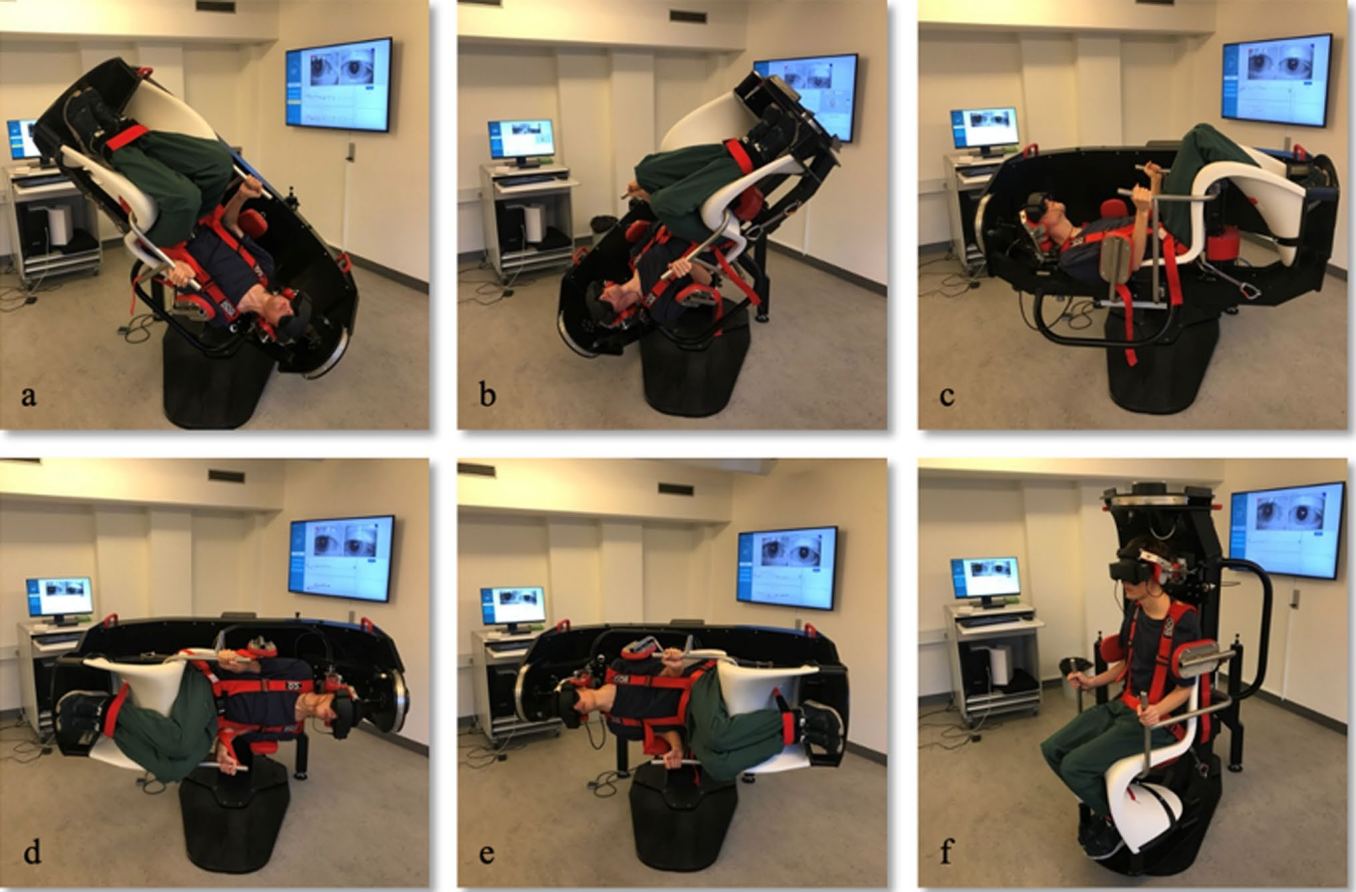
Positional testning with the MRC chair. Dix-Hallpike left (**a**), Dix-Hallpike right (**b**), Supine position (**c**), Supine Roll Test left (**d**), Supine Roll Test right (**e**), Upright position (**f**). The test-setup consisted of (1) a MRC-chair allowing 360 degrees turning in the yaw and pitch axes, (2) VNG-goggles enabling monitoring of eye movements and denying fixation, (3) an operational station (left), and (4) a large TV-screen for optimizing visualization of eye movements (right). All five positions were held for 60 s. Deep DH-test was done by turning the subjects 45 degrees in the yaw axis towards each side in the upright position. Then the subject was moved 135 degrees backwards from the upright position, thereby examining the planes of the vertical SCCs, respectively the left anterior canal along with the right posterior canal (LARP) and the right anterior canal along with the left posterior canal (RALP). The Supine position was reached by tilting the subjects 90 degrees backwards in the pitch plane. The SRT test position was reached by turning the subject 90 degrees in the yaw axis and then tilting the subject 90 degrees to both the left and the right side. Between each of the five positional test positions, subjects were placed in the upright starting position (f). This position was held for 30 s to observe if any nystagmus appeared. The transition time of moving the subject from one position to another was approximately one second

During each examination, subjects were asked to keep their eyes open and to blink as little as possible while looking straight ahead. A cover was used with the VNG-goggles making subjects unable to fixate throughout the entire examination. If a subject was unable to cooperate fully, either because of eye closure or extensive eye blinks, the examination was repeated to ensure the best possible quality of data being collected (Fig. 1).

### Data collection

All data were registered and stored in a secure database (REDCap®, Nashville, Tennessee), that has been approved for secure data management and facilitates secure data collection [12].

Eye movements were tracked with VideoNystagmoGraphy-goggles (VisualEyes™, Interacoustics©, Middelfart, Denmark) via a dark pupil tracking system (OtoAccess*®,* Interacoustics©, Middelfart, Denmark). Eye movements were also visually inspected in real time on a wall-mounted monitor. The actual tracking and registration of eye movements began when the subjects reached the intended positions.

PN was considered to be present if at least five consecutive nystagmus beats with a fast and slow phase in any direction were identified during a five second period. It was registered when the PN started and for how long the PN lasted. Furthermore, PN was described in terms of direction (based upon the fast phase) as either (1) vertical (up- or downbeating), (2) horizontal (left beating or right beating), and (3) torsional (clockwise or counterclockwise as seen from the examiners’ perspective). Intensity of the observed PN was defined as the average Slow-Phase Velocity (a-SPV) measured in degrees per second.

As the accompanying software allows video recording of the observed eye movements, both authors viewed the recordings of eye movements for all subjects following examination to determine if PN was present. During the actual positional testing focus was directed towards patient compliance and the quality of the images (position of the eyes during examination, open eyes and limited eye blinks).

### Statistical analysis

*R* 3.6.0 (Bell Laboratories, Murray Hill, New Jersey) was used for statistical evaluation. Statistics was done by the two authors in cooperation with and guidance of a certified biostatistician.

The prevalence of PN within a healthy population was estimated to be 75% by use of a weighted mean of the prevalence found in seven previously published articles [7, 13–18]. This study aimed to show a difference of 10% compared to the general population. Therefore, we estimated a prevalence of PN of 65% in the study population. When combined with an alpha-value of 0.05 and a power of 80%, a sample size of 156 subjects was needed.

The study was approved by The North Denmark Region Committee on Health Research Ethics (Project-ID N-20220034) and registered at www.ClinicalTrails.gov before inclusion of study subjects was initiated.

## Results

The study population consisted of 78 subjects, 41 women (53%) and 37 men (47%). The mean age of the study population was 28.7 years (SD ± 9.1) with a range from 20 to 57 years.

As a screening method, a vHIT was made to test for any potential vestibular pathology. No pathological saccades were observed, and therefore, no pathological tests were registered. All mean gain-values were within the reference interval.

Of the 78 subjects included in this study, 55 had positional nystagmus (PN) in at least one of the positions examined, resulting in an overall prevalence of 70.5% (Table 1). As shown in Table 1, 55% of subjects presented with PN in more than one examination.

**Table 1:** Prevalence of positional nystagmus

Prevalence (*n* = 78)	*n* (%)
Overall prevalence of positional nystagmus - *Prevalence, females* *- Prevalence, males*	55 (70.5) 29 (70.7)* 26 (70.3)*
Prevalence of PN in multiple test positions	43 (55.0)

^*^Of the total number of participating women and men

A standard error was calculated using the prevalence and number of subjects. Using the standard error, a confidence interval of [0.60;0.81] was calculated. The results of this study therefore suggest that the true prevalence of PN in the general population, with 95% confidence, lies between 60 and 81%.

The definition of PN in this study was observation of a minimum of five nystagmus beats within a five second observation period. The overall prevalence of PN would increase, if definitions of PN similar to other studies were used (Table 2).

**Table 2:** Overall positional nystagmus prevalence with different positional nystagmus criteria

Positional nystagmus criteria	*n* (%)	CI
Minimum of 5 nystagmus beats in 5 consecutive seconds	55 (70.5)	[0.60;0.81]
Minimum of 3 nystagmus beats in 5 consecutive seconds	71 (91.0)	[0.85;0.97]
Minimum of 1 nystagmus beat during the total observation time	77 (98.7)	[0.96;1*]

^*^Truncated from 1.01

Please note that the upper CI-value of “Minimum of 3 nystagmus beats in 5 consecutive seconds” is higher than the lower CI-value of “Minimum of 1 nystagmus beat during the total observation time”, indicating a possibility of the true value being the same for these criteria

Refer to Table 3 for a description of the characteristics of the PN. The majority of observed PN persisted throughout the specific examinations. A more precise notation of the duration of PN was not possible due to the method of recording.

**Table 3:** Description of positional nystagmus

Direction of positional nystagmus (*n* = 55)*	*n* (%)
Vertical, upbeating	45 (81.8)
Vertical, downbeating	7 (12.7)
Horizontal, left beating	17 (30.9)
Horizontal, right beating	25 (45.5)
Torsional, clockwise	2 (3.6)
Torsional, counter clockwise	5 (9.1)

^*^Number of subjects with nystagmus in at least one position

Please note that 81.8% of the subjects presenting with PN had upbeating positional nystagmus in at least one examination

**Fig. 2:**
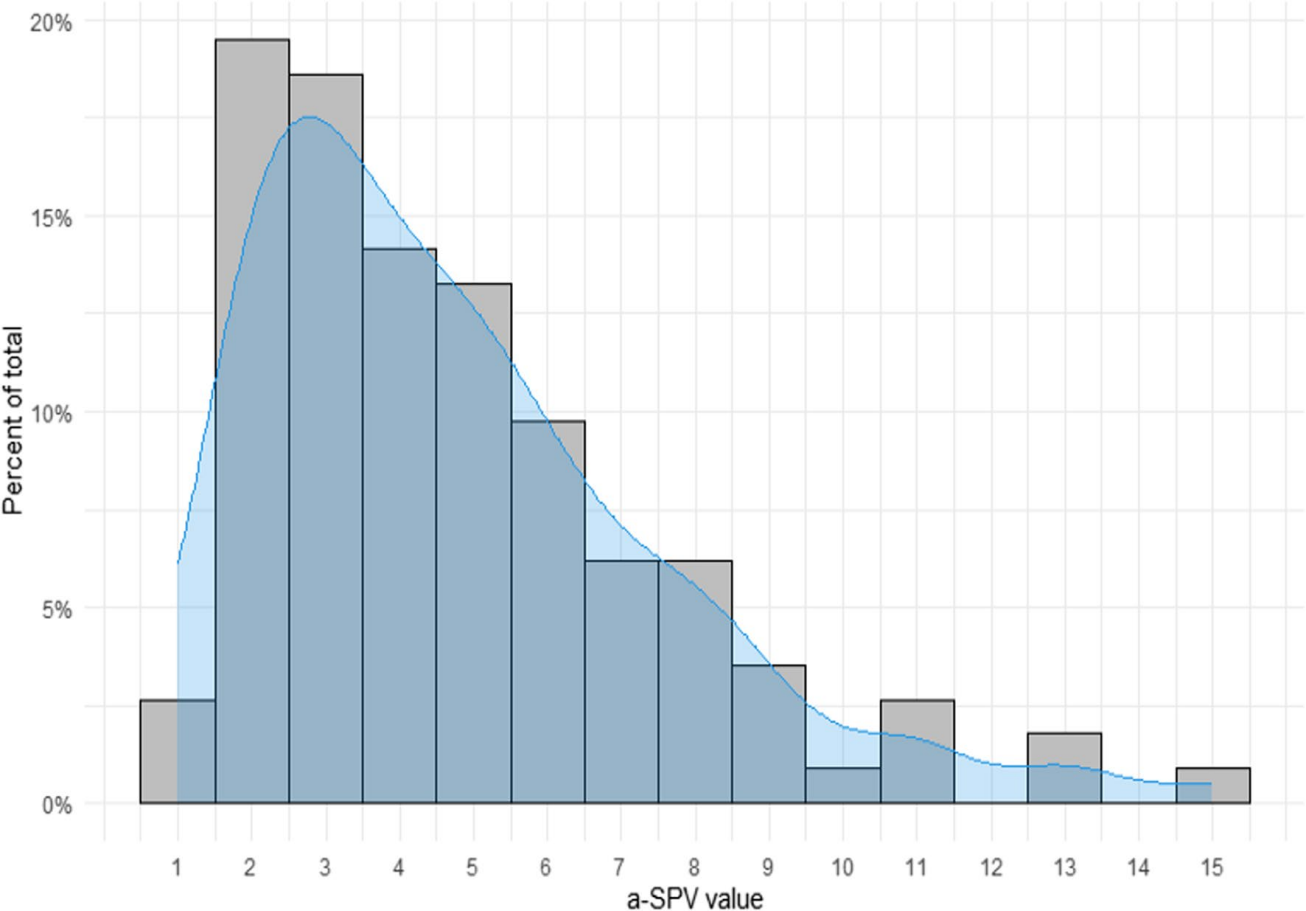
Frequency of individual average Slow Phase Velocity (a-SPV) values. The *x*-axis presents the a-SPV values observed. The *y*-axis shows the frequency of each a-SPV value as a percentage of the total number of a-SPV values. The median a-SPV value was found to be 4 degrees/second, with a 95th percentile of the maximum a-SPV found to be 10.4. The mean a-SPV value was based upon 113 values (67.7% of examinations with PN), as the a-SPV value was not tracked reliably with all examinations where PN was observed

The a-SPV values range from 1 to 15 with a median of 4 and a 95th percentile of 10.4, indicating that 95% of measurements were below 10.4 degrees pr. second (Fig. 2).

Although a few subjects reported mild symptoms (rotational sensation), none were symptomatic similar to patients suffering from BPPV with characteristic positional vertigo. Symptoms were described as a subtle sensation of continuous movement when stopped.

The following table shows the prevalence of PN for each of the five examinations as well as the number of observed PN characteristics that potentially might mimic BPPV (Table 4).

**Table 4:** Prevalence of positional nystagmus clinically relevant to BPPV

Test positions	Direction of positional nystagmus	*n* (%)
Dix-Hallpike, left		29 (52.7)
	Vertical, upbeating	21 (38.2)
	Vertical, downbeating	1 (1.8)
	Torsional, clockwise	0 (0)
	Torsional, counter clockwise	0 (0)
Dix-Hallpike, right		30 (54.5)
	Vertical, upbeating	24 (43.6)
	Vertical, downbeating	2 (3.6)
	Torsional, clockwise	0 (0)
	Torsional, counter clockwise	3 (5.5)*
Supine position		32 (58.2)
	Vertical, upbeating	31 (56.4)
	Vertical, downbeating	0 (0
	Horizontal, left beating	4 (7.3)
	Horizontal, right beating	6 (10.9)
	Torsional, clockwise	0 (0)
	Torsional, counter clockwise	1 (1.8)
Supine Roll Test, left**		23 (41.8)
	Horizontal, left beating	6 (10.9)
	Horizontal, right beating	11 (20.0)
Supine Roll Test, right**		23 (41.8)
	Horizontal, left beating	5 (9.1)
	Horizontal, right beating	10 (18.1)

^*^ All subjects with a torsional PN during a DH-test had either vertical, upbeating (*n* = 2) or vertical downbeating (*n* = 1) PN simultaneously

^**^ 2 subjects had geotropic PN in both SRTs resembling lateral canal BPPV. None had apogeotropic PN in both SRTs

Please note that the examinations of the lateral SCCs (Supine position, SRT left., SRT right.) primarily shows nystagmus not characteristic for lateral BPPV

## Discussion

This study found an overall PN prevalence of 70.5% amongst healthy adults. As mentioned in the methods section, a weighted average of the PN in seven similar studies, weighted by the number of subjects in the individual studies, was estimated to a prevalence of 75%. As 75% is included within the confidence interval of this present study, the estimated prevalence in this study does not differ significantly from similar studies (Table 2) [7, 13–18].

A similar study found the prevalence of PN to be 88% in a study population with a mean age of 40 years [7]. The difference in prevalence could be due to a higher mean age compared to this present study or reflect differences in methodology.

The high prevalence of PN among healthy subjects raises the question whether the current diagnostic criteria are sufficient to ensure an accurate diagnosis of BPPV, especially for inexperienced examiners. Although not all subjects presented with PN mimicking BPPV, some had a relatively high a-SPV and nystagmus resembling BPPV, which could have prompted treatment, if combined with a relevant history of vertigo. This emphasizes the necessity of being careful when diagnosing BPPV, if not all diagnostic criteria are met.

For instance, 82% of subjects with PN showed vertical upbeating PN in at least one position. 53% (CI: [0.40;066]) had vertical upbeating PN in at least one of the two DH-tests, which could imitate the most common subtype and location of BPPV, posterior CAN, and result in unnecessary treatment(s). This result does not differ from a similar study, which found that 41% had vertical, upbeating PN with the DH-tests [7]. However, PN as the authors defined it, is never the sole criteria for BPPV. Concomitant torsional PN is a prerequisite to diagnose BPPV in the vertical SCCs. As this study only found a few healthy subjects presenting with a torsional component in the DH-tests, there is no indication of BPPV being present (Table 4). A limited number of subjects presented with horizontal nystagmus mimicking lateral BPPV in examinations of the lateral SCCs. As a result, the chance of misdiagnosing lateral BPPV is lower.

Table 2 shows that 98.7% had at least one nystagmus beat. This, of course, would not be mistaken as BPPV, but underline nystagmus as being a common finding among healthy adults. Another study defined PN as three beats [19]. As shown, this would have raised the prevalence of PN considerably (Table 2).

In general, the observed PN persisted throughout the examination. This is not typical for BPPV [1]. This implies that the PN observed in healthy adults do not mimic or indicate BPPV of the most common kinds.

Positional nystagmus is predominantly seen with patients that fulfill the diagnostic criteria of BPPV. However, this statement may only be true with patients that have a case history of positional vertigo and therefore undergo standardized testing for BPPV. We acknowledge that, with this study, we encountered a somewhat surprisingly high prevalence of PN in healthy individuals. We used quite strict inclusion and exclusion criteria to make sure that patients included in this study all had normal audio-vestibular function. Despite these rather strict criteria, we did not include normal cerebral imaging as a mandatory prerequisite for inclusion. Theoretically, we may have overlooked minor retro cochlear pathology like e.g., vascular loops. Most studies, however, fail to prove any significant correlation between vascular loops and vestibular paroxysms [20], but this (often unrecognized) condition might theoretically have caused some of the encountered PN. The positional testing with this study was exclusively done by means of an MRC and therefore testing did not include head turns but exclusively whole body turns with DH-testing. We therefore do not believe that vascular compression of e.g., the vertebro-basilar arteries, known to cause vestibular paroxysms during head turns, might have induced PN in this group of healthy individuals.

This study found a median a-SPV of four degrees per second. Furthermore, the 95th percentile of the maximum a-SPV was 10.4 degrees per second. These observations seem to be higher than a similar study [7]. This could be explained by suboptimal tracking during examinations leading to a higher a-SPV than expected but could also be explained by differences in methodology.

When considering all examinations, five subjects (6.4%) were found to have PN mimicking BPPV, e.g., vertical PN with a torsional component during DH-tests or persisting geotropic PN with both SRTs. However, no subjects experienced BPPV characteristic vertiginous symptoms during positional testing.

### Strengths and limitations

A complete vHIT was performed to screen for potential VOR affection of any of the six SCCs. If mean gain-values were above 1.2 (indicating errors during examinations) the test was repeated [11]. It is considered a strength of this study that a thorough vestibular screening, prior to examinations in the T-MRC, was made, as it reduced the possibility of false positives due to e.g., existing inner ear disease.

The T-MRC was used for all examinations. This MRC enables precise and reproduceable positioning with preset 45 degree intervals in the yaw axis and thereby potentially minimizes the intra- and inter examiner variability. MRC usage e.g., the T-MRC, minimizes the effort, and thereby the compliance, required by the subject during positional testing. Concomitant usage of VNG-goggles enables increased monitoring and tracking capabilities of PN. However, the increased visualization of eye movements may result in over diagnostics of BPPV, as examiners may misinterpret normal eye movements as BPPV-related PN.

This study is limited by a selection bias during the recruitment phase. In our case this resulted in recruitment of a specific age group, probably younger than similar studies, which may compromise the inter study comparability. To improve this study, subjects should have been equally distributed in age- and gender specific groups, representing the general population.

The recruitment design caused the mean age of the study population to be noticeably younger than similar studies [7, 15]. This reduces the generalizability, as the study population does not reflect the age distribution of the general population.

Determination and classification of PN in this study was primarily limited to observation and recognition of PN by the two examiners. It allows the authors to accept PN with suboptimal tracking but neglects the objectivity of the software. Although this study aims to determine the prevalence of PN, it is possible that non-quantifiable intra- and inter-examiner differences exist regarding what was considered PN, which would influence the results. To objectify PN, this study had a rather clear definition of PN (five or more consecutive slow- and fast-phase beats in a continuous five second period). Despite optimal visualization of eye movements during positioning, it is feasible that PN may have been overlooked due to excessive eye blinks and/or difficulty of keeping eyes open and still. As the tracking of nystagmus, by using pre-defined software algorithms, has shown to be suboptimal with excessive eye blinks, this was not considered a limitation. The two examiners watched the recordings of every subject’s eye movements together. It could have led to bias as the two examiners might have influenced each other’s conclusion on the presence of PN.

To measure the velocity of nystagmus, and hereby give an estimate of the intensity of nystagmus, the a-SPV was recorded. This is advantageous, as it allows this study to compare its objective findings to other studies. However, due to limitations of the software and reduced patient compliance, it was not always possible to track the subject’s pupils consistently enough, resulting in no/non-reliable a-SPV measures. Therefore, less a-SPV measurements were recorded compared to the total number of examinations with PN, fulfilling the abovementioned criteria, regardless of optimal tracking. Furthermore, the a-SPV measure could potentially be an ambiguous objective value, as there is no guarantee the measured a-SPV reflects the PN fulfilling the criteria of this study.

### Future implications and clinical use

As the possibilities of precise diagnostics advance, the use of more specified diagnostic criteria might be required to ensure less false BPPV-diagnoses and in turn unnecessary treatments, especially to guide inexperienced examiners.

This study underlines the importance of a combined torsional and vertical PN during DH-tests when diagnosing BPPV, as multiple healthy subjects present with purely vertical PN. This is especially important, when examiners have VNG-goggles available due to the improved visualization of eye movements.

Based on this study, an a-SPV value above 10.4 should be considered outside the 95th percentile for healthy adults and an examiner should reconsider potential diagnoses in these cases.

This study advocates for the necessity of a more precise set of diagnostic criteria which should include rotatory vertigo and PN during examinations.

## Conclusion

This study found an overall prevalence of PN of 70.5% within a healthy, adult study population. Therefore, PN may be considered a common finding amongst healthy individuals.

Numerous subjects presented with an upbeating PN. However, only a few had a torsional component concomitantly emphasizing the importance of looking for a combined torsional and vertical PN when diagnosing posterior BPPV. The median a-SPV was 4. Only five subjects (6.4%) presented with PN mimicking BPPV.

This study shows that there is room for optimization of current diagnostic criteria of BPPV. A clear definition of PN (frequency and duration) together with additional parameters like a-SPV might improve BPPV diagnostics. Furthermore, concomitant vertiginous symptoms during examinations should generally be present when diagnosing BPPV.

This study emphasizes the importance of carefully diagnosing BPPV, especially in cases of upbeating PN with no torsional component, a-SPV values below 10.4 degrees per second, patients with no vertiginous symptoms during examinations and no relevant history of rotatory vertigo.

## Data Availability

Not applicable.
